# HGTDR: Advancing drug repurposing with heterogeneous graph transformers

**DOI:** 10.1093/bioinformatics/btae349

**Published:** 2024-06-24

**Authors:** Ali Gharizadeh, Karim Abbasi, Amin Ghareyazi, Mohammad R K Mofrad, Hamid R Rabiee

**Affiliations:** Department of Computer Engineering, Sharif University of Technology, Tehran, P.O. Box 11155-9517, Iran; Department of Computer Engineering, Sharif University of Technology, Tehran, P.O. Box 11155-9517, Iran; Department of Computer Engineering, Sharif University of Technology, Tehran, P.O. Box 11155-9517, Iran; Departments of Bioengineering and Mechanical Engineering, University of California, Berkeley, CA, P.O. Box 94720-1740, United States; Department of Computer Engineering, Sharif University of Technology, Tehran, P.O. Box 11155-9517, Iran

## Abstract

**Motivation:**

Drug repurposing is a viable solution for reducing the time and cost associated with drug development. However, thus far, the proposed drug repurposing approaches still need to meet expectations. Therefore, it is crucial to offer a systematic approach for drug repurposing to achieve cost savings and enhance human lives. In recent years, using biological network-based methods for drug repurposing has generated promising results. Nevertheless, these methods have limitations. Primarily, the scope of these methods is generally limited concerning the size and variety of data they can effectively handle. Another issue arises from the treatment of heterogeneous data, which needs to be addressed or converted into homogeneous data, leading to a loss of information. A significant drawback is that most of these approaches lack end-to-end functionality, necessitating manual implementation and expert knowledge in certain stages.

**Results:**

We propose a new solution, Heterogeneous Graph Transformer for Drug Repurposing (HGTDR), to address the challenges associated with drug repurposing. HGTDR is a three-step approach for knowledge graph-based drug repurposing: (1) constructing a heterogeneous knowledge graph, (2) utilizing a heterogeneous graph transformer network, and (3) computing relationship scores using a fully connected network. By leveraging HGTDR, users gain the ability to manipulate input graphs, extract information from diverse entities, and obtain their desired output. In the evaluation step, we demonstrate that HGTDR performs comparably to previous methods. Furthermore, we review medical studies to validate our method’s top 10 drug repurposing suggestions, which have exhibited promising results. We also demonstrated HGTDR’s capability to predict other types of relations through numerical and experimental validation, such as drug–protein and disease–protein inter-relations.

**Availability and implementation:**

The source code and data are available at https://github.com/bcb-sut/HGTDR and http://git.dml.ir/BCB/HGTDR

## 1 Introduction

The escalating costs and protracted development periods for new pharmaceuticals present significant challenges to the industry (Zhang *et al.* 2020, Pan *et al.* 2022). Drug repurposing, defined as identifying new uses for approved drugs, has emerged as a viable solution to these issues (Zhou *et al.* 2020). Employing this approach mitigates the risk and reduces the expenses and duration associated with the development of drugs (Singh *et al.* 2020, Talevi and Bellera 2020). To facilitate drug repurposing, both systematic experimental and computational techniques have been devised (Pushpakom *et al.* 2019, Talevi and Bellera 2020). Among computational strategies, network-based methods have shown encouraging outcomes, encompassing three primary stages (Zeng *et al.* 2019, Zhu *et al.* 2020, Che *et al.* 2021, Jin *et al.* 2021, Yu *et al.* 2021): (1) network construction, (2) feature extraction, and (3) link prediction. A biological network or knowledge graph is initially constructed, incorporating various biological entities and their interconnections. Subsequently, the nodes within this graph are characterized through embeddings using graph neural networks (GNNs). Finally, these embeddings are applied within algorithms, such as a matrix completion or a neural network, to predict links in the knowledge graph.

Zhu *et al.* (2020) integrated six distinct databases to develop a comprehensive knowledge graph, which comprises five types of entities and nine varieties of relationships. To characterize the graph, they employed a combination of four path-based representations, guided by predefined meta-paths, alongside three embedding-based representations. Subsequently, they harnessed three machine learning techniques—support vector machines, decision trees, and random forests—to discern new potential drug applications. The strength of this study lies in its integration of multiple data sources to account for the interplay between various types of data. Nevertheless, the reliance on manually selected meta-paths may curtail the automation of the method, necessitating specific meta-path selections for each knowledge graph constructed.

The layer attention graph convolutional network (LAGCN) method, as proposed by Yu *et al.* (2021), establishes a drug–drug similarity network by applying the Jaccard index to various drug-related datasets, including targets, pathways, and substructures, all of which are sourced from the DrugBank database. Concurrently, it formulates a disease–disease similarity network, employing semantic similarity scores derived from MeSH descriptors structured as a hierarchical directed acyclic graph (DAG) based on the premise that diseases with more shared ancestral traits exhibit greater similarity. The integration of these two similarity networks, along with the drug–disease relationships, results in the construction of the final input graph. This graph is further processed as nodes are embedded using an LAGCN, followed by the employment of a bilinear decoder for link prediction. While LAGCN leverages diverse data sources, it simplifies these into uniform similarity matrices, a process that may exclude certain informative relationships. Additionally, the method needs more scalability, limiting its applicability to vast graphs.

The AttGCN-DDI model, described by Che *et al.* (2021), assembles a concise knowledge graph from six distinct databases, delineating five entities and seven relationship types. This model hypothesizes that a drug can treat a disease if it can be connected to the disease by a path of less than four steps. Focusing on COVID-19, it incorporates drugs and diseases that have the shortest path to COVID-19 of fewer than four links into the graph. After this addition, the relationships between these newly added nodes are integrated. This method of input selection necessitates expert knowledge in the domain, which, in turn, diminishes the automation aspect of the process. Topological features are extracted as node embeddings using Att-GCN, a method that treats all edges uniformly, thus not accounting for the heterogeneity of the edges. Additionally, a projection matrix is developed to reconstruct the drug–disease interaction matrix. Similar to other discussed methods, this approach also suffers from a lack of scalability.

DeepDR (Zeng *et al.* 2019) employs a network of nine databases to extract drug features. Initially, these networks are transformed into homogeneous similarity networks using techniques such as the Jaccard similarity. The method then generates probabilistic co-occurrence (PCO) matrices for each network through random surfing. To address the inherent sparsity of some networks, it constructs shifted positive pointwise mutual information (PPMI) matrices from the PCO matrices. However, these preprocessing stages may result in losing original network information. A multi-modal deep autoencoder is employed to extract features from drug nodes. DeepDR considers drug–disease associations as inputs and drug features as auxiliary information. Subsequently, it applies a collective variational autoencoder (cVAE), as introduced by Chen and de Rijke (2018), to predict drug–disease associations.

As detailed by Jin *et al.* (2021), HeTDR utilizes an input network resembling the one used by DeepDR. It adopts identical procedures for network conversion to homogeneous networks, random surfing, and the creation of shifted PPMI matrices. A distinctive feature of HeTDR is its application of similarity network fusion to integrate all drug-related information into a singular network, thereby providing a comprehensive biological perspective on drugs.

The model employs a sparse autoencoder, as described in Ng (2011), to extract drug features. Additionally, it utilizes fine-tuned BioBERT (Lee *et al.* 2020) to derive disease features. Consequently, each new input requires the fine-tuning of BioBERT. Ultimately, the GATNE-I model, introduced by Cen *et al.* (2019), is applied to predict drug–disease interactions.

Several contemporary studies have made efforts to utilize heterogeneous data from multiple databases. Nevertheless, these approaches come with constraints. Some, like those proposed by Zhu *et al.* (2020), need more scalability and rely on limited datasets. While methods such as those by Zeng *et al.* (2020) employ different networks, they need to synthesize a higher-order representation that consolidates information from these varied sources. It is crucial to recognize that integrating networks can reveal insights that may need to be discernible when networks are analyzed in isolation. Furthermore, certain techniques (Zeng *et al.* 2019, Yu *et al.* 2021) transform heterogeneous networks into homogenized similarity networks, diluting the input data’s richness. Most of these strategies also do not incorporate non-network data. Some approaches (Zeng *et al.* 2019, Jin *et al.* 2021) restrict the inclusion of certain relationship types in the knowledge graph, thereby preventing the use of pivotal data, such as protein–protein interactions. These methods predominantly center on drug-related data and overlook the extraction of network features pertinent to diseases, thus presenting considerable hurdles to drug repurposing endeavors. Moreover, certain methods necessitate hands-on implementation, including the designation of meta-paths as specified by Zhu *et al.* (2020), necessitating specialized domain knowledge and constraining the potential for input modification. The absence of end-to-end model structures further compromises the automation of these methods and amplifies the reliance on domain expertise.

To address the constraints outlined previously, we introduce a model endowed with the following features:

- It distinguishes between node and edge types using heterogeneous graphs.
- The model accommodates an unrestricted variety of graph data types, enabling the use of any node and edge categories in the input graph.
- Scalability is a core attribute of the method.
- The model operates autonomously, obviating the need for domain-specific knowledge during setup.
- It is an end-to-end model that integrates the task of link prediction within the feature extraction process from nodes.

Our model, Heterogeneous Graph Transformer for Drug Repurposing (HGTDR), parallels prior models by encompassing a three-step approach. In our research, PrimeKG (Chandak *et al.* 2023) serves as the foundation of our knowledge graph, complemented by the initial embeddings from BioBERT (Lee *et al.* 2020) and ChemBERTa (Chithrananda *et al.* 2020). To extract node features, we utilize the heterogeneous graph transformer (HGT) technique (Hu *et al.* 2020) as our GNN. Finally, a fully connected network predicts the relationship score within the graph between a drug and a disease.

PrimeKG significantly enhances the initial phase of our approach, broadening the scope of drugs and diseases that can be explored beyond previous capabilities. Table 1 outlines a comparative analysis of the coverage of drugs and diseases by this method and others.

**Table 1.** Comparison of the number of drugs and diseases using different methods.

Method	Drug	Disease
ATTGCN-DDI	1470	752
LAGCN	269	598
HetDR	1519	1229
DeepDDR	1519	1229
HGTDR	1801	1363

Several studies have developed knowledge graphs in drug repurposing, including Hetionet (Himmelstein *et al.* 2017) and DRKG (Ioannidis *et al.* 2020). Hetionet features 755 indication relations, linking 387 drugs to 77 diseases. DRKG presents 83 895 compound–disease relationships, which may not directly pertain to indications. Notably, most of these relationships, specifically 77 782, are derived from the GNBR database (Percha and Altman 2018), which utilizes text-processing techniques. However, this source may not reliably inform drug repurposing efforts due to its reliance on text extraction.

For the extraction of features from heterogeneous graphs, various methodologies have been proposed, such as HAN (Wang *et al.* 2019), HetGNN (Zhang *et al.* 2019), MHGCN (Yu *et al.* 2022), and HGT (Hu *et al.* 2020). HAN employs node-level and semantic-level attention mechanisms within heterogeneous graphs to discern the significance of nodes and their meta-path-based neighbors and the relevance of different meta-paths. HetGNN introduces a strategy for capturing the structural and content heterogeneity. It utilizes a random walk approach to sample heterogeneous neighbors that are strongly correlated, grouping them by node type. Subsequently, node embeddings are generated through a dual-module neural network that accounts for structural and content diversity. MHGCN aims to autonomously identify valuable heterogeneous meta-path interactions across varying lengths in multiplex heterogeneous networks through multi-layer convolutional aggregation, thereby producing node embeddings that merge structural and semantic information. Despite its innovations, MHGCN’s scalability remains a challenge. HGT distinguishes itself by interpreting heterogeneous nodes and edges through meta-relations, utilizing node-specific and edge-specific weights. This approach enables identifying common and unique patterns across different relationships without predefined meta-paths, thus bypassing concerns about identifying critical meta-paths within the biological context.

According to the study by Hu *et al.* (2020), HGT outperforms HAN and HetGNN, further validated by its scalability advantage. Therefore, we have selected HGT for node feature extraction in our model. While the original study by Hu *et al.* highlighted HAN’s effectiveness following HGT, we opted to verify HGT’s superiority by substituting our feature extraction layers with HAN layers. The results in Table 4 affirm that HGT surpasses HAN in our specific task.

**Table 4.** Comparison of HGT and HAN layers as feature extractors.

Feature extraction layer	AUROC	AUPR
HAN	0.925	0.918
HGT	0.944	0.946

The rest of this article is organized as follows: Section 2 is dedicated to a comprehensive exposition of our methodology. Section 3 presents the experiments conducted and the corresponding results obtained through the application of our method. The paper concludes in Section 4, where we provide concluding remarks and outline directions for future research.

## 2 Materials and methods

This section begins with the presentation of the problem formulation, followed by a detailed explanation of the three steps involved in our proposed method.

### 2.1 Problem formulation

Let G be a heterogeneous graph whose nodes are biological entities whose edges are different relationships among the entities. We have drug and disease nodes and indication edges in the graph. We define all indications as $I={\left\{\left(Dr^{i},\;Di^{i}\right),\;Y^{i}\right\}}_{i=1}^{N}$ where $\left(Dr^{i},Di^{i}\right)$ is the i^th^ indication edge in the graph; $Y^{i}$ is the label, which is 1 for all indications, and N is the number of all indications. During the training step, we put 20% of indications ($I_{\mathrm{input}}$) in the graph G and tried to predict the label of the rest of the indications ($I_{\mathrm{pos}}$) plus random negative samples, which are defined as $\mathrm{Ineg}\;=\;{\left\{\left(Dr^{i},\;Di^{j}\;\right),\;0\right\}}_{1}^{N_{\mathrm{pos}}}$ where $N_{\mathrm{pos}}$ is the number of samples of $I_{\mathrm{pos}}$, $i\in (1\ldots N_{\mathrm{Drug}})$, and $j\in (1\ldots N_{\mathrm{Disease}})$. $N_{\mathrm{Drug}}$ and $N_{\mathrm{Disease}}$ are also the number of drugs and diseases in G, respectively.

(1) $$I_{\mathrm{prediction}}=I_{\mathrm{pos}}+I_{\mathrm{neg}}$$

This prediction task can be viewed as a simulation of drug repurposing in which our model learns to predict indications that do not exist in the input graph. To do actual drug repurposing, we must put all indications in the graph G and try to predict novel indications.

### 2.2 Network construction

In this step, we should construct a network. Previous methods built their networks using databases such as DrugBank (Law *et al.* 2014). There is a problem with this approach. Domain knowledge is necessary to select the appropriate databases. Moreover, not all relations within a database are used in those works, raising the question of how the relations are chosen. We address this problem by utilizing a previously constructed knowledge graph, PrimeKG (Chandak *et al.* 2023), and allowing our model to determine which entities and relationships are relevant to the task. To achieve this goal, HGT is suitable because it can control the overall contribution of a particular node or edge type to embedding creation by using node and edge-specific weights. PrimeKG integrates 20 high-quality resources to generate a graph with 10 node types and 30 edge types. The existing graph can also be expanded with new data without node and edge type restrictions. The graph is defined as G=(V, E, A, R) in which every node v∈V and every edge e∈E are associated with their type-mapping functions $\tau \left(v\right):\;V\to A$ and $\phi \left(e\right):E\to R$ respectively. Also, for every edge e=(s, t), where s and t are the source and target nodes of the edge, its meta-relation is denoted as ⟨$\tau \left(s\right),\;\phi \left(e\right),\;\tau (t)\rangle$.

Since previous works only contain drugs and diseases that contribute to indication relations, we remove drug and disease nodes that do not contribute to at least one indication edge. These nodes can increase accuracy unrealistically, in comparison with previous works, due to the ease of predicting no indications related to the drugs and diseases. Consequently, some nodes are removed from the graph. Tables 2 and 3 demonstrate graph statistics before and after the removal of the nodes.

**Table 2.** PrimeKG node counts.

Entity	Count before removal	Count after removal	Removal percent
Biological process	28 642	28 642	0
Protein	27 671	27 573	0.35
Disease	17 080	1363	92.01
Phenotype	15 311	15 082	1.49
Anatomy	14 035	14 035	0
Molecular function	11 169	11 169	0
Drug	7957	1801	77.36
Cellular component	4176	4176	0
Pathway	2516	2516	0
Exposure	818	780	4.64
**Total**	**129** **375**	**107** **137**	**17.17**

**Table 3.** PrimeKG directed edge counts.

Entity	Count before removal	Count after removal	Removal percent
Anatomy–protein (present)	3 036 406	3 036 406	0
Drug–drug	2 672 628	743 328	72.18
Protein–protein	642 150	642 150	0
Disease–phenotype (positive)	300 634	24 490	91.85
Biological process–protein	289 610	289 610	0
Cellular component–protein	166 804	166 804	0
Disease–protein	160 822	99 232	38.29
Molecular function–protein	139 060	139 060	0
Drug–phenotype	129 568	102 736	20.70
Biological process–biological process	105 772	105 772	0
Pathway–protein	85 292	85 292	0
Disease–disease	64 388	1772	97.24
Drug–disease (contraindication)	61 350	32 194	47.52
Drug–protein	51 306	24 848	51.56
Anatomy–protein (absent)	39 774	39 774	0
Phenotype–phenotype	37 472	37 472	0
Anatomy–anatomy	28 064	28 064	0
Molecular function–molecular function	27 148	27 148	0
Drug–disease (indication)	18 776	18 776	0
Cellular component–cellular component	9690	9690	0
Phenotype–protein	6660	6660	0
Drug–disease (off-label use)	5136	3836	25.31
Pathway–pathway	5070	5070	0
Exposure–disease	4608	2924	36.54
Exposure–exposure	4140	4140	0
Exposure–biological process	3250	3250	0
Exposure–protein	2424	2424	0
Disease–phenotype (negative)	2386	140	94.13
Exposure–molecular function	90	90	0
Exposure–cellular component	20	20	0
**Total**	**8** **100** **498**	**5** **683** **172**	**29.84**

#### 2.2.1 Initial embeddings

We add BioBERT and ChemBERTa embeddings to our knowledge graph to integrate different types of information into the graph. BioBERT is a domain-specific language representation model pre-trained on large-scale biomedical corpora. Therefore, its embeddings add information extracted from biomedical literature to our knowledge graph. ChemBERTa is a similar model pre-trained on SMILES (Weininger 1988) representation of molecules that can extract their structural information. The initial embeddings are added to the graph in the following manner. The names of all entities, except drugs, are first obtained from PrimeKG. BioBERT embeddings are then extracted and added to our graph nodes. Moreover, SMILES (Weininger 1988) representations of the drugs are obtained from DrugBank. Afterward, they are embedded using ChemBERTa and added to drug nodes. Interestingly, we can add any embedding to any of our node types, and we do not require different node types to have similar embeddings. This lets us add any auxiliary information to our graph if it can be represented as an embedding. We denote the initial embeddings of the graph as $H^{\mathrm{init}}$.

After constructing the graph, we divide indication edges into masked and unmasked groups, with 80% of indication edges being masked. Unmasked indications are used like other edges as part of the input graph. However, masked indications are used as positive samples when computing loss function. This technique helps the model predict indications not present in the input graph, which can act like a simulation of drug repurposing.

### 2.3 Feature extraction

For embedding graph nodes, attention-based GNNs usually follow the following formula for source node s and target node t (Hu *et al.* 2020):

(2) $$H^{l}\left[t\right]\leftarrow \underset{\forall s\in N\left(t\right),\forall e\in E\left(s,t\right)}{\mathrm{Aggregate}}\left(\mathrm{Attention}\left(s,t\right).\mathrm{Message}\left(s\right)\right)$$

There are three basic operators: Attention, which estimates the importance of each source node; Message, which extracts the message using only the source nodes; and Aggregate, which aggregates the neighborhood message by the attention weight.

To understand different distributions of different node and edge types, HGT introduces a heterogeneous mutual attention mechanism to calculate a target node and all its neighbors (s∈N(t)) mutual attention grounded by their meta-relation. The attention for each edge $e=\left(s,t\right)$ is defined as follows:

$$\mathrm{Attentio}n_{\mathrm{HGT}}\left(s,e,t\right)=\underset{\forall s\in N\left(t\right)}{\mathrm{Softmax}}\left(\underset{i\in \left[1,\;h\right]}{\|}A\mathrm{TT}—\mathrm{hea}d^{i}\left(s,e,t\right)\right)$$

$$\mathrm{ATT}—\mathrm{hea}d^{i}\left(s,e,t\right)=\left(K^{i}\left(s\right)W_{\Phi \left(e\right)}^{\mathrm{ATT}}Q^{i}{\left(t\right)}^{T}\right).\frac{\mu_{<\tau \left(s\right),\;\Phi \left(e\right),\tau \left(t\right)>}}{\sqrt{d}\;}$$

$$K^{i}\left(s\right)=K—\mathrm{Linea}r_{\tau \left(s\right)}^{i}\left(H^{\left(l-1\right)}\left[s\right]\right)$$

(3) $$Q^{i}\left(t\right)=Q—\mathrm{Linea}r_{\tau \left(t\right)}^{i}\left(H^{\left(l-1\right)}\left[t\right]\right)$$

For the i-th attention head $\mathrm{ATT}—\mathrm{hea}d^{i}(s,\;e,\;t)$, source nodes that have type τ(s) is projected into the i-th key vector $K^{i}\left(s\right)$, with a node-specific linear projection to consider the distribution differences of node types. Similarly, the target node t is projected to the i-th query vector. Furthermore, unlike the vanilla Transformer that directly computes the dot product between query and key vectors for calculating the similarity between key and query, HGT keeps a distinct edge-specific matrix $W_{\Phi \left(e\right)}^{\mathrm{ATT}}$ for each edge type $\Phi \left(e\right)$. Thus, the model can capture different semantic relations even between the same node-type pairs. Additionally, a prior tensor μ is used to denote the general significance of each meta-relation triplet, serving as an adaptive scaling to the attention. Finally, h attention heads are concatenated to get each node pair’s attention vector. Then, for each target node t, all attention vectors of neighbors N(t) are gathered, and softmax is conducted, making it fulfill $\Sigma_{\forall s\in N\left(t\right)}\mathrm{Attentio}n_{\mathrm{HGT}}\left(s,e,t\right)=1_{h\times 1}$.

Similarly, meta-relations of edges are also used in message passing process to alleviate the distribution differences of nodes and edges of different types as follows:

$$\mathrm{Messag}e_{\mathrm{HGT}}\left(s,e,t\right)=\underset{i\in \left[1,\;h\right]}{\|}M\mathrm{SG}—\mathrm{hea}d^{i}\left(s,e,t\right)$$

(4) $$\mathrm{MSG}—\mathrm{hea}d^{i}\left(s,e,t\right)=M—\mathrm{Linea}r_{\tau \left(s\right)}^{i}\left(H^{\left(l-1\right)}\left[s\right]\right)W_{\Phi \left(e\right)}^{\mathrm{MSG}}$$

Source node’s ID with a linear projection $M—\mathrm{Linea}r_{\tau \left(s\right)}^{i}$. It is then followed by a matrix $W_{\Phi \left(e\right)}^{\mathrm{MSG}}$ for incorporating the edge dependency. Then, all h message heads are concatenated for each node pair.

In the aggregation step, the attention vector is used as the weight to average the corresponding messages from the source nodes to get the updated target vector ${\hat{H}}^{\left(l\right)}[t]$ as:

(5) $${\tilde{H}}^{\left(l\right)}\left[t\right]=\underset{\forall s\in N\left(t\right)}{⊕}\left(\mathrm{Attentio}n_{\mathrm{HGT}}\left(s,e,t\right).\mathrm{Messag}e_{\mathrm{HGT}}\left(s,e,t\right)\right)$$

Finally, the target node’s vector is mapped back to its type-specific distribution with a linear projection $A—\mathrm{Linea}r_{\tau \left(t\right)}$ followed by a non-linear activation and residual connection as:

(6) $$H^{\left(l\right)}\left[t\right]=\sigma \left(A—\mathrm{Linea}r_{\tau \left(t\right)}{\tilde{H}}^{\left(l\right)}\left[t\right]\right)+H^{\left(l-1\right)}[t]$$

As described, the method relies on using meta-relation to parameterize the weight matrices separately, enabling the model to distinguish the operators for different relations and thus be more capable of handling the distribution differences in heterogeneous graphs.

#### 2.3.1 HGSampling

Using the entire input graph in the training process can make any method unscalable. Therefore, a sampling method is needed to make the method scalable. Consequently, we use HGSampling (Hu *et al.* 2020) to get mini-batches for training. We denote a subgraph generated by the sampler as $G_{\mathrm{batch}}$. The method keeps a similar number of nodes and edges for each type and keeps the subgraph dense to minimize information loss and reduce sample variance. HGSampling has been used in large-scale graphs and applied to a graph with 178 million nodes and 2.2 billion edges (Hu *et al.* 2020). As a result, there will not be any problem regarding the size of the graph since the current large biological graphs have fewer than 10 million edges (Himmelstein *et al.* 2017, Zhang *et al.* 2021, Chandak *et al.* 2023).

#### 2.3.2 Model architecture

Our model has three layers of HGT (L=3) whose input and output feature vector sizes are 64. The initial embeddings are 768 in size, which are the outputs of the BioBERT and ChemBERTa models. Therefore, a linear layer is first defined, which decreases the input size to 64.

(7) $$H_{\mathrm{batch}}^{0}=\mathrm{Dropout}\left(\mathrm{ReLU}\left(\mathrm{Linea}r_{\tau }\left(H_{\mathrm{batch}}^{\mathrm{init}}\right)\right)\right)$$

where $H_{\mathrm{batch}}^{\mathrm{init}}$ is the input feature vector containing the initial embedding of batch nodes, $H_{\mathrm{batch}}^{0}$ is the input feature vector of the first HGT layer, $\mathrm{Linea}r_{\tau }$ is a node-specific linear layer, and ReLU is the rectified linear unit activation function. Also, dropout is applied to the output with probability = 0.5.

HGT layers are defined as follows:

(8) $$H_{\mathrm{batch}}^{l}=\mathrm{HGT}(H_{\mathrm{batch}}^{\left(l-1\right)},\;{\;G}_{\mathrm{batch}})$$

where $G_{\mathrm{batch}}$ is the sampled input graph, and $H_{\mathrm{batch}}^{\left(l-1\right)}\;$and $H_{\mathrm{batch}}^{l}\;$are the input and output feature vectors of layer l. All layers have eight HGT attention heads like the original work on HGT (Hu *et al.* 2020). The outputs of all three layers are concatenated and fed to the following linear layer.

(9) $$\mathrm{Feat}=\mathrm{ReLU}\left(\mathrm{Linear}\left(i\in \left[i,\;L\right]{||H}_{\mathrm{batch}}^{i}\right)\right)$$

where Feat is the output feature vector for subgraph nodes.

### 2.4 Link prediction

Link prediction tasks are carried out with a simple two-layer fully connected network, which receives input from a concatenated collection of drug and disease feature vectors derived from the HGT network. Suppose we want to check the score of the drug i and disease j in our input subgraph. We first concatenate HGT embeddings of the drug and the disease:

(10) $$H_{\mathrm{FC}}^{0}=\mathrm{Fea}t_{\mathrm{Dru}g_{i}}\;||\;\mathrm{Fea}t_{\mathrm{Diseas}e_{j}}$$

where $\mathrm{Fea}t_{\mathrm{Dru}g_{i}}$ and $\mathrm{Fea}t_{\mathrm{Diseas}e_{j}}$ are extracted features for drug i and disease j, respectively. The first layer’s input dimension size is 128 (concatenation of two 64-dimensional embeddings), and the output dimension size is 64. Batch normalization is used in this layer, ReLU is the activation function, and the dropout rate is 0.2.

(11) $$H_{\mathrm{FC}}^{1}=\mathrm{Dropout}\left(\mathrm{ReLU}\left(\mathrm{BatchNorm}\left(\mathrm{Linea}r_{128\to 64}\left(H_{\mathrm{FC}}^{0}\right)\right)\right)\right)$$

**Figure 1.**
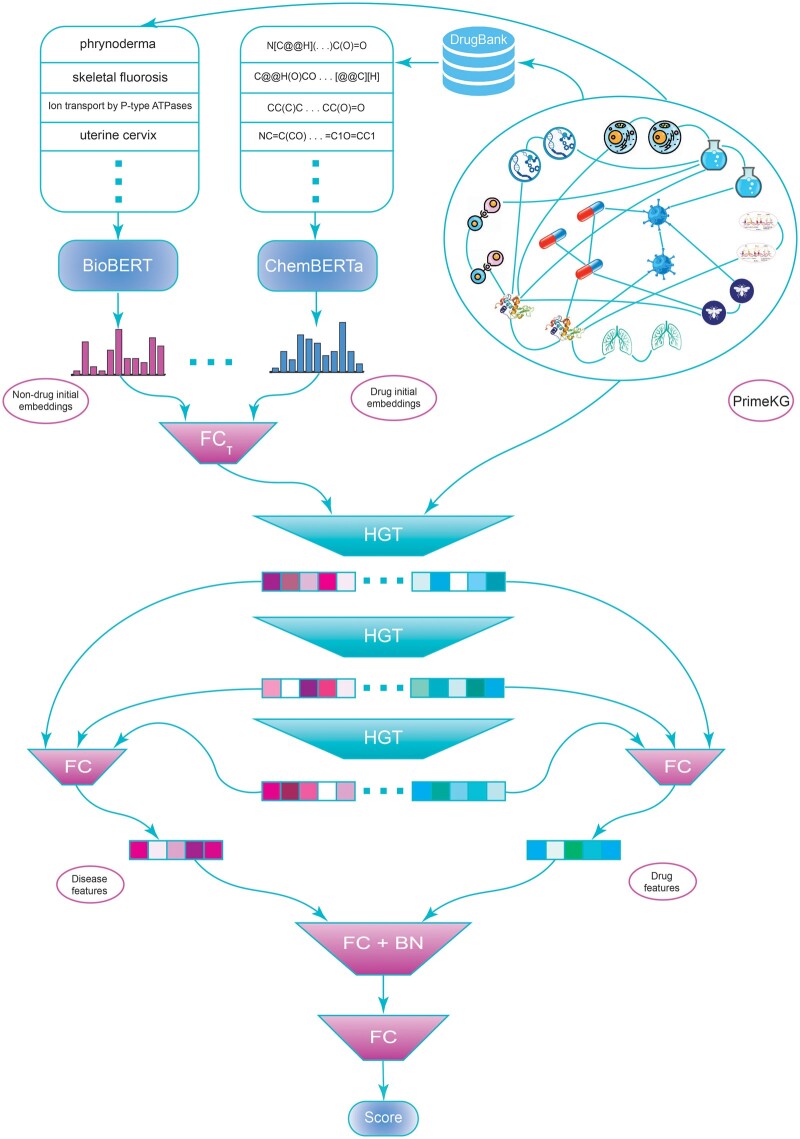
(1) PrimeKG is obtained as the knowledge graph, (2) drug SMILES representations are extracted from DrugBank, and entity names are extracted from PrimeKG, (3) initial embeddings are extracted using BioBERT and ChemBERTa, (4) node embeddings are computed using HGT layers, (5) final embeddings are obtained with applying a fully connected layer on a concatenation of node embeddings of different layers, and (6) drug-disease relation score is computed using a 2-layer FC network.

The second layer is as follows:

(12) $$\mathrm{Score}=\sigma \left(\mathrm{Linea}r_{64\to 1}\left(H_{\mathrm{FC}}^{1}\right)\right)$$

where σ is the sigmoid function, and Score is the output of a fully connected network, which is the indication score between drug i and disease j. Figure 1 demonstrates each step of the method in detail.

### 2.5 Training

The graph is generated, as explained in Section 2.2. During training, subgraphs are generated by the HGSampling algorithm using drugs as initial sample nodes. The batch size of sampling is 164 because we have 1801 drugs in our graph, and we want our subgraphs to be similar in size. And other batch sizes can let one batch have very few drugs. HGSampling produces a sampled sub-graph of L depth from the initial nodes when the sampling depth is L. As we employ three HGT layers in the model, embeddings are constructed from neighboring nodes within a distance of three nodes. Therefore, we chose to have a sampling depth of three. As demonstrated in Table 6, similar results are obtained when different sampling depths (L = 2, 3, or 4) are used; however, sampling depths of 2 and 3 perform slightly better than depths of 4. Also, the number of nodes sampled in each iteration for each node type is 512. The HGT network and link-predicting network are trained as an end-to-end model.

**Table 6.** Comparing HGSampling with different sampling depths.

Sampling depth	AUROC	AUPR
2	0.946	0.945
3	0.944	0.946
4	0.943	0.944

We use the binary cross-entropy function as the model’s loss function on $I_{\mathrm{predictio}n_{\mathrm{batch}}}$ which contains the positive and negative samples for $G_{\mathrm{batch}}$.

(13) $$\mathrm{Loss}=-\frac{1}{n}\sum_{i=1}^{n}Y_{i}\;.\;\log\left({\hat{Y}}_{i}\right)+\left(1-Y_{i}\right)\;.\;\log(1-{\hat{Y}}_{i})$$

where n is the number of all samples, $Y_{i}$ is the label of the i^th^ sample, and ${\hat{Y}}_{i}$ is the output of the model for the i^th^ sample. Positive samples are masked indications of the subgraph, and negative samples are randomly selected from all possible edges between the subgraph’s drugs and diseases, which are not actually in the subgraph’s masked or unmasked indications.

The model has been optimized via the AdamW (Loshchilov and Hutter 2019) optimizer with a cosine annealing learning rate scheduler (Loshchilov and Hutter 2017). The model is trained for 300 epochs.

## 3 Experiments

Our experiments and their results are explained in this section. We compared HGTDR with LAGCN (Yu *et al.* 2021), DeepDR (Zeng *et al.* 2019), and HeTDR (Jin *et al.* 2021) using 5-fold cross-validation to evaluate the proposed method. The results for all methods were regenerated for this work. AUROC and AUPR are used as evaluation metrics for comparison.

Hyperparameter optimization is also done on the percent of masked indications (20%, 50%, 80%), number of HGT layers (3, 4), and layer feature space dimension (32, 64). The model is run using an NVIDIA GeForce GTX 1080 Ti GPU with a memory size of 11GB.

As described in the introduction, we compared the effectiveness of HGT layers with HAN layers to show their effectiveness. Furthermore, to show the result’s insensitivity to the sampling method, we tested different sampling depths and repeated validation with different samplings.

In Section 2.2, we claim that drug and disease nodes should be removed to make our data comparable to previous works, and using those removed nodes can increase our method’s performance unrealistically. In an effort to validate this fact, we carried out an experiment where the nodes were not removed.

We conducted another experiment to compare HeTDR and HGTDR’s performance for new diseases, where literature and indication information are limited. In this experiment, we used 90% of diseases for training and 10% for testing. We removed BioBERT embeddings for diseases of each method, and only one indication from every validation disease was used in training data. Since one of HeTDR’s limitations is that it does not support validating diseases that do not indicate training data.

Furthermore, we ran another experiment to check the robustness of our model to different input types. In this experiment, we ran our model five times, and each time, we removed one type of relation in the input graph (five edge types with most instances are used).

In addition, to investigate the model’s ability to extract the necessary information for its task, we use it without any modifications to predict other relations instead of indications. Input data and model are both the same in this experiment.

### 3.1 Results

**Figure 2.**
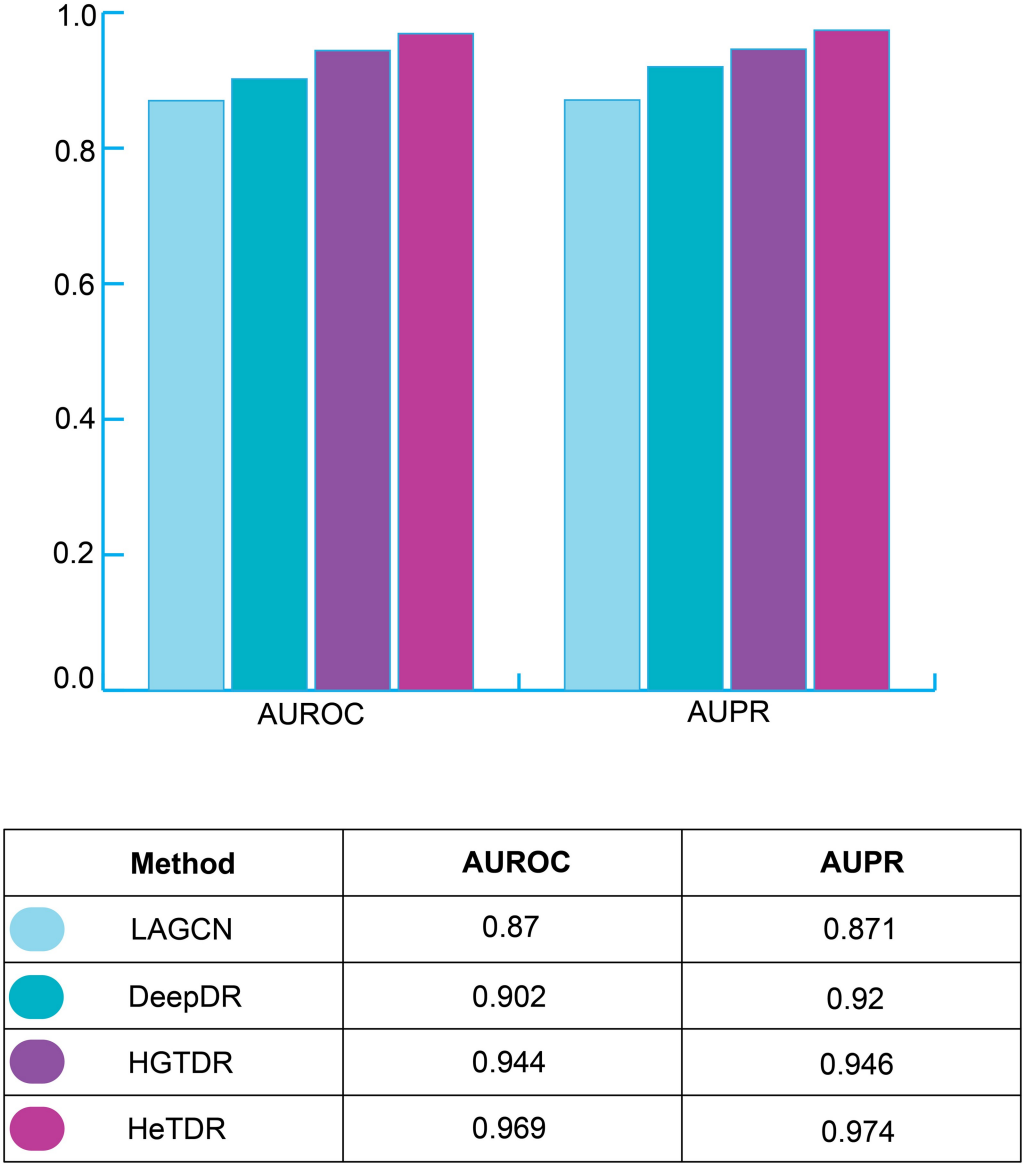
Comparing AUROC and AUPR metrics for LAGCN, DeepDR, HeTDR, and HGTDR.

For cross-validation, we divided indications into five subsets. Four subsets were used for training the model, and one subset was used for validation in each fold. For each metric, the average of 5-fold is reported. Figure 2 shows the comparison results of the four methods. Only HeTDR performs slightly better than our method.

We showed the effectiveness of HGT layers by swapping them with HAN layers. Table 4 demonstrates the higher performance of HGT layers in comparison with HAN layers.

Additionally, we demonstrated that different samplings using the HGSampling algorithm do not lead to much difference in prediction results. Table 5 shows validation results for 10 repeats on one cross-validation fold with different samplings.

**Table 5.** Validation results for 10 repeats on 1-fold of cross-validation.

AUROC	AUPR
0.951	0.954
0.950	0.952
0.949	0.950
0.951	0.953
0.950	0.951
0.950	0.953
0.951	0.954
0.945	0.947
0.950	0.951
0.954	0.958

Moreover, Table 6 shows that the method is not sensitive to sampling depth and performs well when using 2 or 3 sampling depths.

Table 7 investigates the effect of drug and disease nodes’ removal, as discussed in Section 2.2. We can see that not removing those nodes increases the method’s performance. However, since other methods do not use such drug and disease nodes where there is no indication related, we decided not to use those nodes in our main method.

**Table 7.** HGTDR performance compared with when no node is removed from the input graph.

Method	AUROC	AUPR
HGTDR	0.944	0.946
HGTDR without node removal	0.979	0.970

In a second experiment designed for new diseases, we compared HGTDR and HeTDR. Table 8 demonstrates the results of the two methods with the data limitations of new diseases.

**Table 8.** HGTDR and HeTDR results without literature and indication information.

Method	AUPR	AUROC
HGTDR	0.859	0.871
HeTDR	0.779	0.782

As explained in Section 3, cross-validation was repeated for each of the five robustness testing experiments. Table 9 demonstrates the results of robustness testing of HGTDR to input variation. There is no significant difference in the results of the six types of inputs, which shows that HGTDR is robust to relation removals. Notably, more than half of the edges of our original graph are in anatomy-protein type, and removing the type does not have much effect on the results. Any relationship contains valuable information for drug repurposing or can be considered noise in the input graph. Using this method, we do not need to know which relation type is proper and which is noise for our task.

**Table 9.** The method results when a relation type is removed from the input graph.

Removed relation	AUPR	AUROC
Nothing removed	0.946	0.944
Anatomy–protein(present)	0.949	0.949
Drug–drug	0.945	0.944
Protein–protein	0.944	0.943
Biological process–protein	0.940	0.942
Cellular component–protein	0.939	0.939

#### 3.1.1 Novel relation prediction

Although common metrics like AUROC and AUPR show the general correctness of these methods, they are not entirely reliable since the ultimate goal of drug repurposing is to find false positive examples. Hence, a method with an accuracy of 100% would not be helpful because it cannot suggest novel drug repurposing candidates. To predict novel indications, we trained the model with all indications and made 110 batches (10 sets of batches with batch size 164) from the input graph with positive and negative samples. False positive predictions can be inferred as drug repurposing candidates. We further investigated medical literature to find evidence for novel relations predicted by HGTDR. Table 10 demonstrates discovered evidence from the literature for 10 highly scored false positive predictions by HGTDR.

**Table 10.** Experimental evidence of novel predicted relations.

Drug	Disease	Evidence
Betamethasone	Lichen planus	(Cawson 1968)
Dactinomycin	Acute myeloid leukemia	(Falini *et al.* 2015)
Vincristine	Ewing sarcoma	(Wagner *et al.* 2017)
Paclitaxel	Classic Hodgkin lymphoma	(Sinha *et al.* 2013)
Prednisolone	Trichinellosis	(Shimoni *et al.* 2007)
Dexamethasone	Blastomycosis	No evidence
Vinblastine	Malignant Sertoli-Leydig Cell tumor of the ovary	(Finan *et al.* 1992)
Escitalopram	Social phobia	(Pelissolo 2008)
Dactinomycin	Plasmablastic lymphoma	No evidence
Cetirizine	Urticaria	(Kalivas *et al.* 1990)

#### 3.1.2 Other applications

Even though our primary goal in developing this method was to predict drug–disease links, its generality enables us to use it to predict any relationship in the graph. Thus, we used it to predict other relation types. Table 11 shows evaluation results for those relations.

**Table 11.** Evaluation results when predicting relations other than indication.

Relation	AUPR	AUROC
Disease–protein	0.912	0.926
Drug–protein	0.951	0.948
Pathway–protein	0.951	0.953
Drug–phenotype	0.885	0.911
Biological process–protein	0.889	0.888
Protein–protein	0.881	0.887

We also investigated other research to evaluate these predictions experimentally. Tables 12 and 13 show the experimental validation for disease–protein and drug–protein relations. Medical literature supports five out of five disease–protein relation predictions and three out of five drug–protein relation predictions. Experimental and numerical evidence suggests that the model can extract the information it needs to complete its task without changing the inputs or providing additional information.

**Table 12.** Experimental evidence of novel disease–protein relations.

Protein	Disease	Evidence
VEGFA	Lynch syndrome	(Tjalma *et al.* 2016)
CCND1	Acute lymphoblastic leukemia	(Hsu *et al.* 2021)
MTHFR	Carcinoma of esophagus	(Wang *et al.*, 2008)
IL6	Scleroderma	(Feghali *et al.* 1992)
IL1B	Hepatitis	(Hirankarn *et al.* 2006)

**Table 13.** Experimental evidence of novel drug–protein relations.

Protein	Drug	Evidence
OPRM1	Clozapine	(Solismaa *et al.* 2018)
SHBG	Fostamatinib	No evidence
UGT1A8	Acetaminophen	(Yoshizawa *et al.* 2020)
DRD5	Pentobarbital	No evidence
CYP1A1	Amitriptyline	(Mayer *et al.* 2007)

To determine which information from the graph is most relevant to each downstream task, we used meta-relation attention to identify the most important relations for each task. Supplementary Tables S1–S7, in supplementary materials, present the top five meta-relations for all three layers in our model for different tasks. For indication prediction, we can see that other relations between drugs and diseases, like contraindication and off-label use, are among the important meta-relations, which is logical. Furthermore, we can see that most of the important meta-relations in all tables have a common node type with the meta-path that is being predicted. For example, in protein–protein prediction, all important meta-paths have node types of protein in them.

## 4 Discussion

Drug repurposing is a promising strategy to overcome current drug development limitations such as high failure risk and long development time. Therefore, many works have tried to develop systematic experimental and computational drug repurposing methods. Some previous works suggested methods that limit input data in different ways to increase numerical evaluation performance or fit the data to their models. However, numerical evaluation cannot be fully trusted in this task since a method’s lower accuracy is probably due to suggesting more actual drug repurposing candidates. Hence, manual data manipulation may unnoticeably hinder a method’s drug-repurposing ability.

This work presents an end-to-end, automatic method that removes previous works’ data limitations while maintaining comparable performance. This method can use any graph data type, even with massive scales, plus embedded side information to predict novel indications. We do not force the method to utilize some parts of the data with a greater emphasis. Nevertheless, the model learns which input part is relevant to the task. As a result of this feature, the method also performs well on other tasks, such as predicting drug–protein and disease–protein relationships.

A three-step method for drug repurposing is proposed using a heterogeneous input graph that overcomes some of the data-related limitations of previous studies. It is important to note that data limitations can make a method incapable of helping in the case of new disease outbreaks such as COVID, in which only limited information is available regarding the disease. In the first step, network construction, we attempt to use an existing graph rather than create a new one instead of previous works. As a result, we are preventing the pipeline from being biased by our domain knowledge. Additionally, we avoid any further preprocessing, such as constructing similarity matrices, which is another source of bias, and allow our method to determine how information should be extracted from the input data. We only remove drug and disease nodes that do not contribute to any indication edge to make the results comparable to previous works. It is noteworthy that removing the nodes adversely impacts our performance, which aligns with expectations since it is easy for the model to predict no indication relating to those nodes. Our method could add any embedded information to graph entities. Therefore, we add BioBERT and ChemBERTa embeddings to the graph, providing literature and drug structure information. In the second step, heterogeneous graph transformer layers extract node features. The architecture of HGT can extract implicit meta-paths of the graph and capture the common and specific patterns of different relationships. Due to these properties, we can use any entity and relations in the input with side information without manipulating them to increase performance. As high-throughput technologies make vast amounts of data available in various areas, scalable drug repurposing has become increasingly important. Thus, we use HGSampling to make the method scalable as well. Lastly, we use a fully connected neural network end-to-end with the previous steps to customize the extracted features for the downstream task. This enables the method to be employed for other similar tasks, too.

A 5-fold cross-validation procedure was utilized for evaluation, and the AUPR and AUROC metrics were compared with three other state-of-the-art methods. The method named HeTDR performed better than HGTDR by about 2% in both measures with their default conditions. However, HGTDR outperformed HeTDR by about 8% when new disease conditions are assumed in both methods. These metrics, however, cannot demonstrate the same performance since we cannot evaluate all the false positive samples that may be unknown true positives. We examined the experimental evidence for this case’s top 10 false positives. Eight out of 10 predictions had evidence from literature suggesting an indication relationship. We also found that our method was robust to input variation when some relations from the input graph were removed. To show the applicability of HGTDR in other tasks, we changed the downstream task of indication prediction to six other tasks without modifying the model. Both numerical and experimental evaluations suggest that the model can extract informative features when the task is shifted.

Our method facilitates adding information to a knowledge graph and predicting hitherto unidentified information. Nonetheless, the extension of medical data encounters constraints, notably due to the lack of unique identifiers for entities, which hampers the addition of fresh information. While there is a potential to map and convert identifiers, the process is not exhaustive, with some data remaining unmapped and hence unconverted. Another limitation inherent in our method is its focus on predicting relationships numerically without the capacity to substantiate these predictions with empirical evidence. For instance, although our method can predict whether a drug is suitable for treating a disease, it cannot elucidate the drug’s mechanism of action. Additionally, this method does not allow us to add side information to edges like we do for nodes. It is important to note that some information does not relate to a node but rather to an edge. As an example, the expression of a gene in a disease is information related to an edge that this approach cannot use.

In future research, there is the opportunity to enrich the knowledge graph by expanding the existing graph structure and incorporating varied types of ancillary information from emergent sources. Given the multifaceted nature of drug development, a holistic assimilation of information could substantially enhance performance. Additionally, the methodology has potential applications in combination therapies, which could benefit from the integrated embeddings of multiple drugs. Furthermore, efforts can be directed toward augmenting the interpretability of the end-to-end framework, thus increasing the utility and understanding of the predictive outcomes.

## Supplementary Material

btae349_Supplementary_Data

## Data Availability

Our code and data are available at: https://github.com/bcb-sut/HGTDR; http://git.dml.ir/BCB/HGTDR.
