# Supplementary material for: HGTDR: Advancing drug repurposing with heterogeneous graph transformers
Source: Bioinformatics. 2024 Jun 24;40(7):btae349. doi: 10.1093/bioinformatics/btae349 (PMC11223801; doi:10.1093/bioinformatics/btae349)
Supplement: btae349_Supplementary_Data [file btae349_supplementary_data.docx]

**HGTDR: Advancing Drug Repurposing with Heterogeneous Graph Transformers**

**Supplementary Materials**

**Table S1. Important meta-relations for indication prediction**

| **Layer** | **Node type** | **Edge type** | **Node type** | **Meta-relation importance** |
| --- | --- | --- | --- | --- |
| Layer 3 | Disease | Contraindication | Drug | 1.306 |
|  | Disease | Disease-disease | Disease | 1.287 |
|  | Drug | Off-label use | Disease | 1.174 |
|  | Protein | Drug-protein | Drug | 1.122 |
|  | Phenotype | Drug-phenotype | Drug | 1.091 |
| Layer 2 | Disease | Disease-disease | Disease | 1.252 |
|  | Protein | Phenotype-protein | Phenotype | 1.226 |
|  | Disease | Contraindication | Drug | 1.201 |
|  | Disease | Off-label use | Drug | 1.193 |
|  | Phenotype | Disease-phenotype (positive) | Disease | 1.147 |
| Layer 1 | Disease | Off-label use | Drug | 1.178 |
|  | Disease | Disease-disease | Disease | 1.136 |
|  | Protein | Drug-protein | Drug | 1.121 |
|  | Protein | Phenotype-protein | Phenotype | 1.102 |
|  | Exposure | Exposure-protein | Protein | 1.079 |

**Table S2. Important meta-relations for disease-protein prediction**

| **Layer** | **Node type** | **Edge type** | **Node type** | **Meta-relation importance** |
| --- | --- | --- | --- | --- |
| Layer 3 | Disease | Disease-disease | Disease | 1.347 |
|  | Cellular component | Cellular component-protein | Protein | 1.229 |
|  | Protein | Protein-protein | Protein | 1.229 |
|  | Biological process | Biological process-protein | Protein | 1.217 |
|  | Phenotype | Phenotype-protein | Protein | 1.208 |
| Layer 2 | Protein | Molecular function-protein | Molecular function | 1.467 |
|  | Disease | Disease-disease | Disease | 1.305 |
|  | Phenotype | Phenotype-protein | Protein | 1.296 |
|  | Protein | Biological process-protein | Biological process | 1.249 |
|  | Protein | Protein-protein | Protein | 1.242 |
| Layer 1 | Disease | Disease-disease | Disease | 1.293 |
|  | Pathway | Pathway-protein | Protein | 1.228 |
|  | Disease | Off-label use | Drug | 1.202 |
|  | Disease | Indication | Drug | 1.195 |
|  | Phenotype | Disease-phenotype (positive) | Disease | 1.182 |

**Table S3. Important meta-relations for drug-protein prediction.**

| **Layer** | **Node type** | **Edge type** | **Node type** | **Meta-relation importance** |
| --- | --- | --- | --- | --- |
| Layer 3 | Protein | Protein-protein | Protein | 1.292 |
|  | Disease | Disease-protein | Protein | 1.213 |
|  | Phenotype | Phenotype-protein | protein | 1.183 |
|  | Cellular component | Cellular component-protein | Protein | 1.146 |
|  | Drug | Drug-drug | Drug | 1.138 |
| Layer 2 | Protein | Molecular function-protein | Molecular function | 1.427 |
|  | Protein | Biological process-protein | Biological process | 1.316 |
|  | Disease | Indication | drug | 1.247 |
|  | Phenotype | Phenotype-protein | Protein | 1.198 |
|  | Pathway | Pathway-protein | Protein | 1.184 |
| Layer 1 | Disease | Indication | Drug | 1.182 |
|  | Protein | Molecular function-protein | Molecular function | 1.175 |
|  | Pathway | Pathway-protein | Protein | 1.174 |
|  | Biological process | Biological process-protein | Protein | 1.160 |
|  | Phenotype | Disease-phenotype (positive) | Disease | 1.133 |

**Table S4. Important meta-relations for pathway-protein prediction.**

| **Layer** | **Node type** | **Edge type** | **Node type** | **Meta-relation importance** |
| --- | --- | --- | --- | --- |
| Layer 3 | Cellular component | Cellular component-protein | Protein | 1.239 |
|  | Biological process | Biological process-protein | Protein | 1.207 |
|  | Protein | Protein-protein | Protein | 1.177 |
|  | Molecular function | Molecular function-protein | Protein | 1.120 |
|  | Drug | Drug-protein | Protein | 1.118 |
| Layer 2 | Pathway | Pathway-pathway | Pathway | 1.449 |
|  | Protein | Protein-protein | Protein | 1.320 |
|  | Cellular component | Cellular component-protein | Protein | 1.219 |
|  | Protein | Biological process-protein | Biological process | 1.208 |
|  | Disease | Disease-protein | Protein | 1.204 |
| Layer 1 | Pathway | Pathway-pathway | Pathway | 1.459 |
|  | Protein | Protein-protein | Protein | 1.208 |
|  | Biological process | Biological process-protein | Protein | 1.179 |
|  | Phenotype | Phenotype-protein | Protein | 1.175 |
|  | Disease | Disease-protein | Protein | 1.131 |

**Table S5. Important meta-relations for drug-phenotype prediction.**

| **Layer** | **Node type** | **Edge type** | **Node type** | **Meta-relation importance** |
| --- | --- | --- | --- | --- |
| Layer 3 | Drug | Drug-drug | Drug | 1.194 |
|  | Protein | Phenotype-protein | Phenotype | 1.152 |
|  | Disease | Disease-phenotype (positive) | Phenotype | 1.129 |
|  | Protein | Drug-protein | Drug | 1.118 |
|  | Phenotype | Phenotype-phenotype | Phenotype | 1.077 |
| Layer 2 | Phenotype | Phenotype-phenotype | Phenotype | 1.373 |
|  | Protein | Drug-protein | Drug | 1.140 |
|  | Disease | Disease-phenotype (positive) | Phenotype | 1.124 |
|  | Exposure | Exposure-protein | Protein | 1.104 |
|  | Drug | Drug-drug | Drug | 1.090 |
| Layer 1 | Phenotype | Phenotype-phenotype | Phenotype | 1.292 |
|  | Disease | Disease-phenotype (positive) | Phenotype | 1.196 |
|  | Pathway | Pathway-protein | Protein | 1.073 |
|  | Disease | Contraindication | Drug | 1.072 |
|  | Protein | Drug-protein | Protein | 1.067 |

**Table S6. Important meta-relations for biological process-protein prediction.**

| **Layer** | **Node type** | **Edge type** | **Node type** | **Meta-relation importance** |
| --- | --- | --- | --- | --- |
| Layer 3 | Biological process | Biological process-biological process | Biological process | 1.345 |
|  | Disease | Disease-protein | Protein | 1.281 |
|  | Protein | Protein-protein | Protein | 1.224 |
|  | Drug | Drug-protein | Protein | 1.221 |
|  | Exposure | Exposure-protein | Protein | 1.219 |
| Layer 2 | Protein | Protein-protein | Protein | 1.413 |
|  | Biological process | Biological process-biological process | Biological process | 1.402 |
|  | Protein | Drug-protein | Drug | 1.368 |
|  | Disease | Disease-protein | Protein | 1.238 |
|  | Phenotype | Phenotype-protein | Protein | 1.220 |
| Layer 1 | Biological process | Biological process-biological process | Biological process | 1.516 |
|  | Pathway | Pathway-protein | Protein | 1.226 |
|  | Phenotype | Phenotype-protein | Protein | 1.150 |
|  | Exposure | Exposure-biological process | Biological process | 1.147 |
|  | Protein | Protein-protein | Protein | 1.132 |

**Table S7. Important meta-relations for protein-protein prediction.**

| **Layer** | **Node type** | **Edge type** | **Node type** | **Meta-relation importance** |
| --- | --- | --- | --- | --- |
| Layer 3 | Pathway | Pathway-protein | Protein | 1.370 |
|  | Drug | Drug-protein | Protein | 1.296 |
|  | Phenotype | Phenotype-protein | Protein | 1.281 |
|  | Disease | Disease-protein | Protein | 1.273 |
|  | Cellular component | Cellular component-protein | Protein | 1.252 |
| Layer 2 | Phenotype | Phenotype-protein | Protein | 1.383 |
|  | Biological process | Biological process-protein | Protein | 1.320 |
|  | Anatomy | Anatomy-protein (absent) | Protein | 1.316 |
|  | Pathway | Pathway-protein | Protein | 1.314 |
|  | Exposure | Exposure-protein | Protein | 1.288 |
| Layer 1 | Pathway | Pathway-protein | Protein | 1.360 |
|  | Phenotype | Phenotype-protein | Protein | 1.299 |
|  | Biological process | Biological process-protein | Protein | 1.293 |
|  | Anatomy | Anatomy-protein (absent) | Protein | 1.269 |
|  | Disease | Disease-protein | Protein | 1.232 |
